# Evaluating Outcome Prediction via Baseline, End-of-Treatment, and Delta Radiomics on PET-CT Images of Primary Mediastinal Large B-Cell Lymphoma

**DOI:** 10.3390/cancers16061090

**Published:** 2024-03-08

**Authors:** Fereshteh Yousefirizi, Claire Gowdy, Ivan S. Klyuzhin, Maziar Sabouri, Petter Tonseth, Anna R. Hayden, Donald Wilson, Laurie H. Sehn, David W. Scott, Christian Steidl, Kerry J. Savage, Carlos F. Uribe, Arman Rahmim

**Affiliations:** 1 Department of Integrative Oncology, BC Cancer Research Institute, Vancouver, BC V5Z 1L3, Canada; ivan.skn@outlook.com (I.S.K.); maziars@student.ubc.ca (M.S.); curibe@bccrc.ca (C.F.U.); arahmim@bccrc.ca (A.R.); 2 BC Children’s Hospital, Vancouver, BC V6H 3N1, Canada; claire.gowdy@cw.bc.ca; 3 Department of Physics and Astronomy, University of British Columbia, Vancouver, BC V6T 1Z1, Canada; 4 Division of Radiology, BC Cancer, Vancouver, BC V5Z 1L3, Canada; pete.tonseth@bccancer.bc.ca (P.T.); dwilson@bccancer.bc.ca (D.W.); 5 Centre for Lymphoid Cancer, BC Cancer, Vancouver, BC V5Z 1L3, Canada; anna.hayden1@bccancer.bc.ca (A.R.H.); lsehn@bccancer.bc.ca (L.H.S.); dscott8@bccancer.bc.ca (D.W.S.); csteidl@bccancer.bc.ca (C.S.); ksavage@bccancer.bc.ca (K.J.S.); 6 Department of Pathology and Laboratory Medicine, University of British Columbia, Vancouver, BC V6T 1Z7, Canada; 7 BC Cancer, Vancouver, BC V5Z 1L3, Canada; 8 Department of Radiology, University of British Columbia, Vancouver, BC V5Z 1M9, Canada; 9 Department of Biomedical Engineering, University of British Columbia, Vancouver, BC V6T 2B9, Canada

**Keywords:** ^18^F-FDG PET/CT, Primary Mediastinal Large B-cell Lymphoma, progression prediction, machine learning, radiomics, time to progression

## Abstract

**Simple Summary:**

This study aims to evaluate the feasibility of using changes in radiomic features over time (Delta radiomics) following chemotherapy to predict relapse/progression and time to progression (TTP) in primary mediastinal large B-cell lymphoma (PMBCL) patients. Analyzing data from 103 PMBCL patients, including end-of-treatment (EoT) scans and longitudinal radiomics features, the study employed various machine learning techniques for prediction. Results indicate that using Delta radiomics improves prediction accuracy for relapse/progression and TTP compared to using only EoT radiomics features. The study underscores the importance of EoT scans and the potential of Delta radiomics in predicting disease progression in PMBCL patients based on [^18^F]FDG PET-CT scans.

**Abstract:**

Objectives: Accurate outcome prediction is important for making informed clinical decisions in cancer treatment. In this study, we assessed the feasibility of using changes in radiomic features over time (Delta radiomics: absolute and relative) following chemotherapy, to predict relapse/progression and time to progression (TTP) of primary mediastinal large B-cell lymphoma (PMBCL) patients. Material and Methods: Given the lack of standard staging PET scans until 2011, only 31 out of 103 PMBCL patients in our retrospective study had both pre-treatment and end-of-treatment (EoT) scans. Consequently, our radiomics analysis focused on these 31 patients who underwent [^18^F]FDG PET-CT scans before and after R-CHOP chemotherapy. Expert manual lesion segmentation was conducted on their scans for delta radiomics analysis, along with an additional 19 EoT scans, totaling 50 segmented scans for single time point analysis. Radiomics features (on PET and CT), along with maximum and mean standardized uptake values (SUVmax and SUVmean), total metabolic tumor volume (TMTV), tumor dissemination (Dmax), total lesion glycolysis (TLG), and the area under the curve of cumulative standardized uptake value-volume histogram (AUC-CSH) were calculated. We additionally applied longitudinal analysis using radial mean intensity (RIM) changes. For prediction of relapse/progression, we utilized the individual coefficient approximation for risk estimation (ICARE) and machine learning (ML) techniques (K-Nearest Neighbor (KNN), Linear Discriminant Analysis (LDA), and Random Forest (RF)) including sequential feature selection (SFS) following correlation analysis for feature selection. For TTP, ICARE and CoxNet approaches were utilized. In all models, we used nested cross-validation (CV) (with 10 outer folds and 5 repetitions, along with 5 inner folds and 20 repetitions) after balancing the dataset using Synthetic Minority Oversampling TEchnique (SMOTE). Results: To predict relapse/progression using Delta radiomics between the baseline (staging) and EoT scans, the best performances in terms of accuracy and F1 score (F1 score is the harmonic mean of precision and recall, where precision is the ratio of true positives to the sum of true positives and false positives, and recall is the ratio of true positives to the sum of true positives and false negatives) were achieved with ICARE (accuracy = 0.81 ± 0.15, F1 = 0.77 ± 0.18), RF (accuracy = 0.89 ± 0.04, F1 = 0.87 ± 0.04), and LDA (accuracy = 0.89 ± 0.03, F1 = 0.89 ± 0.03), that are higher compared to the predictive power achieved by using only EoT radiomics features. For the second category of our analysis, TTP prediction, the best performer was CoxNet (LASSO feature selection) with c-index = 0.67 ± 0.06 when using baseline + Delta features (inclusion of both baseline and Delta features). The TTP results via Delta radiomics were comparable to the use of radiomics features extracted from EoT scans for TTP analysis (c-index = 0.68 ± 0.09) using CoxNet (with SFS). The performance of Deauville Score (DS) for TTP was c-index = 0.66 ± 0.09 for n = 50 and 0.67 ± 03 for n = 31 cases when using EoT scans with no significant differences compared to the radiomics signature from either EoT scans or baseline + Delta features (*p*-value> 0.05). Conclusion: This work demonstrates the potential of Delta radiomics and the importance of using EoT scans to predict progression and TTP from PMBCL [^18^F]FDG PET-CT scans.

## 1. Introduction

Primary Mediastinal Large B-cell Lymphoma (PMBCL) is a distinct lymphoma type with global incidence of 0.4 per million [1,2]. PMBCL typically has a favorable outcome following rituximab based chemotherapy. Nevertheless, despite the initial treatment, approximately 5–10% of patients will experience refractory disease, which is associated with poor outcomes [1,3]. Most cases of recurrence occur within two years and may include extra nodal sites [4,5,6,7]. The International Conference on Malignant Lymphomas Working Group has released guidelines recommending the optimized use of PET/CT for staging and assessing the response of lymphomas, utilizing the five-point Deauville scale (DS) [8].

Several quantitative imaging parameters have been evaluated that may predict response to therapy, ranging from tumor volume-based metrics to metabolic characteristics like shape and texture. Total metabolic tumor volume (TMTV) has been found to be a significant predictor of outcome in lymphoma [9,10,11,12,13,14,15,16]. However, TMTV is not quantified in clinical reports due to the time-consuming and labor-intensive nature of tumor segmentation. To bridge this gap, we proposed an AI-powered automated segmentation approach tailored for lymphoma PET/CT images, utilizing a cascaded framework [17]. Additionally, there are conflicting findings across different datasets regarding the predictive power of TMTV [18,19] and SUVmax [20,21]. Ceriani et al. [22] showed that glucose consumption via Total Lesion Glycolysis (TLG) and Metabolic Heterogeneity (MH) demonstrated superior accuracy in identifying high-risk patients prone to disease progression. There are a number of studies that investigated the feasibility of employing PET “radiomics”, to predict treatment outcomes in PMBCL patients [3]. The term radiomics was first introduced in 2010 [23], and subsequently was established in 2012 as a systematic process using machine learning (ML) [24]. This process involves extracting a wide range of quantitative metrics (such as shape, intensity, filter-based, or textural features) in a high-throughput manner from radiological images [25,26]. Several studies assessed the value of higher-order textural features in predicting overall survival for patients with diffuse large B-cell lymphoma (DLBCL), as well as for various other tumor types [27,28,29,30].

Radiomics signatures extracted from medical images typically rely on single-time scans, ignoring changes during treatment. Delta radiomics, capturing changes in features over time, offers potential in evaluating treatment response. ML with delta radiomics outperforms single-time-point radiomics in predicting outcomes, promising improved clinical decision-making [31]. However, there is a growing interest in “Delta radiomics,” which involves analyzing features changes over time (longitudinal) to predict outcomes [32,33,34,35,36,37,38,39,40,41] with increased robustness compared to single time-point radiomics features [42,43]. Delta radiomics also is favored due to routine imaging in cancer diagnosis and follow-up, promoting its utilization in clinical settings [44]. To emphasize the changes of the radiomics signature due to therapy two definitions of Delta radiomics have been considered in this study, namely the absolute difference [45] or the relative difference [46]. The present work utilizes both staging (pre-therapy or baseline) and post-therapy (end-of-treatment (EoT)) [^18^F]FDG PET/CT scans as the two time-points for Delta radiomics analysis. (For the rest of this paper we use the baseline and EoT terms).

The scope of our investigation in this study covers a patient group which spans 10 years and treated with rituximab based anthracycline-containing chemotherapy. We hypothesized that there is a difference between some of the features on the baseline and EoT studies following a standard course of chemotherapy for PMBCL that can serve as a biomarker of treatment failure. In the following, we investigate the effectiveness of using Delta radiomics by applying individual coefficient approximation for risk estimation (ICARE) [47] and ML techniques to predict progression and time to progression (TTP).

## 2. Materials and Methods

### 2.1. Patient Data

Imaging files of 103 patients diagnosed with PMBCL from 2005 to 2021 and treated with curative intent chemoimmunotherapy (R-CHOP) were retrospectively analyzed with eligibility criteria of a diagnosis of PMBCL following the review of tumor histology centrally by an expert pathologist and clinical review to confirm a predominant anterior mediastinal mass as per WHO classification [3]. Scans were performed on two scanners (Discovery 690 PET/CT and Discovery 600 PET/CT; GE Healthcare). All patients were managed according to BC Cancer protocol which states a 6 h fast and sampled blood glucose of <200 ng/dL prior to the injection of 300–400 MBq [^18^F]FDG followed by a 60 min uptake phase. Ethics approval for this study was received by the Research Ethics Board at BC Cancer. Until 2011, an EoT PET scan was performed in all patients, but due to limited resources, staging PET scans were not standard. Out of 103 PMBCL patients, only 31 had both pre-treatment and EoT scans due to resource constraints. Hence, we focused on these 31 patients for Delta radiomics analysis, as they underwent [^18^F]FDG PET-CT scans before initiating therapy and after completing a 6-cycle course of R-CHOP chemotherapy.

For a comprehensive radiomics analysis, accurate lesion segmentation by expert (nuclear medicine) physicians is crucial. Expert manual lesion segmentation was performed on these 31 pre-treatment scans and their corresponding EoT scans. Furthermore, manual segmentation was extended to encompass an additional 19 EoT scans from other cases, bringing the total number of segmented EoT scans to 50. This approach allowed for a larger set of cases to be included in the single time-point radiomics analysis, enhancing the comprehensiveness of the study findings (Table 1). EoT scans patients with Deauville scores of D1–3 are classified as achieving complete metabolic response versus partial metabolic response (D4/D5) or progressive disease (D5—new sites or progression of original sites). It is noteworthy to highlight that, in this study, the determination of relapse/progression is reliant on information obtained from follow-up scans.

### 2.2. Manual Segmentation

The nuclear medicine physician conducted multiple segmentations on PET images, utilizing 41% SUVmax, 25% SUVmax, manual segmentation, and PET-Edge via the MIM software (version 7.2.3). In previous studies [48], we established minimal inter-observer variability and high intra-class correlation with PET-Edge MIM segmentation. Consequently, we employed this segmentation method for the analysis in the current study. The segmentations were performed blind to patient outcome. Though PET and CT images for each patient are hardware-registered during acquisition via a hybrid PET/CT scanner, to perform CT segmentation, we performed additional PET-CT registration via affine registration.

### 2.3. Extraction of Radiomics Features and Computation of Delta Radiomics

**Figure 1:**
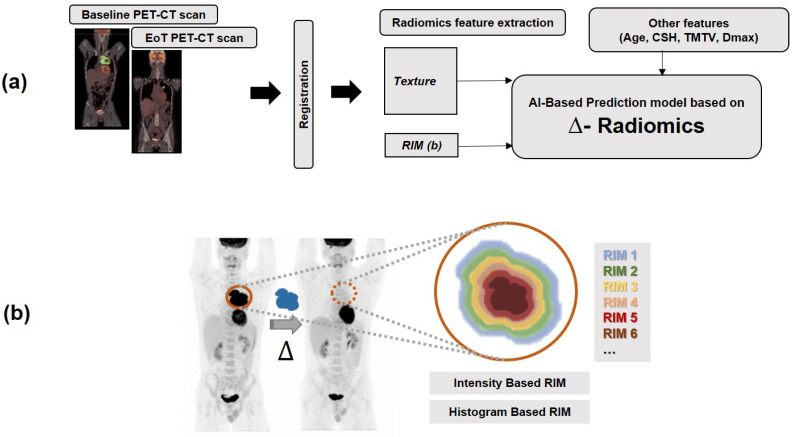
Our suggested approach: (**a**) Diagrammatic representation of Radiomics flow chart applied in this study. (**b**) RIM features are extracted on scans from different time points (baseline and End of Treatment (EoT)) on both PET and CT scans (Only PET scan is shown in (**b**) for visualization). (RIM: radial mean intensity).

The workflow representation of this study is shown in Figure 1a. Intensity and textural features were extracted with the Pyradiomics package [49]. First-order radiomic features recorded were the SUVmax, SUVmean, metabolic tumor volume (MTV), TLG and entropy of the large mediastinal mass. Higher order radiomic features of Neighbourhood Grey Tone Difference Matrix (NGTDM) including Busyness, Coarseness, Complexity, Contrast, Strength, and the Grey Level Run Length Matrix (GLRLM) encompassing Grey Level Nonuniformity and Run Length Non-uniformity were also extracted. The number of grey levels was 64 for PET and 400 for CT, and the bin size was 0.3125 for PET (SUV units), and 10 Hounsfield Units (HU) for CT [50]. CSH (intensity-based area under the Curve) were also added to the features.

The segmentation contour of the primary tumor volume on the baseline scan was deformably registered to the EoT scan [34] (Figure 1a). In this study, two definitions of Delta radiomics have been used: (i) The absolute change in each radiomic feature following treatment is determined by subtracting the baseline feature values from the EoT values [45]:

(1) $${∆}_{Radiomics\_absolute}=\left|{{EoT}_{Radiomics}-Baseline}_{Radiomics}\right|$$

(ii) The relative changes of each radiomic feature due to treatment is calculated as follows [46]:

(2) $${∆}_{Radiomics\_relative}=\frac{\left|{{EoT}_{Radiomics}-Baseline}_{Radiomics}\right|}{{Baseline}_{Radiomics}}$$

Despite the robust discriminative capabilities showcased by Delta radiomics, which focus on longitudinal changes in radiomic features over the entire lesion area, these methods often emphasize temporal variations. In contrast, Radial Intensity Mean (RIM) introduces a perspective by representing the average intensity value across successive layers of voxels (envelope). These layers extend from the outermost region to the innermost, with each layer having a thickness of one voxel. As the envelopes progressively reduce in size (via 3D erosion of one voxel), they move towards the center of the region of interest (ROI) [51,52] (Figure 1b). We considered intensity-based and histogram-based conventional features including minimum intensity, mean intensity, standard deviation of intensity and maximum intensity. We also considered the number of voxels, an approximation of volume and sum of the intensities in each shell. However, we did not use the ideas of sphere-shell partition [53] in this study since, due to the irregular shape of tumors, each sphere-shell region can encompass both intratumoral and peritumoral regions.

#### 2.3.1. Missing Data Imputation

To address missing data, i.e., NaN (Not a Number) values for features, the priority between accuracy and speed, data normalization, and the extent of missing data should be considered. For faster processing, simple statistical methods such as use of mean or median values are possible as they offer a good balance between accuracy and speed. However, if the emphasis is on maximizing accuracy, more advanced techniques such as K-Nearest Neighbors (KNN) (especially for low percentages of missing data) should be taken into account [54]. The choice ultimately depends on the specific requirements and trade-offs desired in the given scenario. In this work, we used the mean imputation approach.

#### 2.3.2. Harmonization

Initially, we need to employ statistical testing to determine the presence of scanner additive effects (indicating a shift in mean) and multiplicative effects (indicating heteroscedasticity) in the data obtained from two scanners at different time points, that are specifically utilized for our Delta radiomics analysis (GE Discovery 600 and 690). Two-sample *t*-test and Bartlett’s test were used, respectively for mean shift (additive effect) and heteroscedasticity (multiplicative effect) assessment. For the Delta radiomics study, since we have PET-CT imaging data for a specific patient at two time points and these two scans were acquired with different scanners in some cases, we concluded to use longitudinal ComBat [55]. The results of the additive and multiplicative effect tests and longitudinal harmonization are presented in the Supplementary Material (Harmonization effect).

### 2.4. Prediction Tasks

In this study, we aim to predict relapse/progression and TTP using PET and CT radiomics features. We focus on evaluating the prognostic power of Delta radiomics. We analyze various feature sets, including baseline, EoT, Relative Delta, and Absolute Delta, as well as combinations such as baseline + EoT. Additionally, we explore including baseline and Delta, both relative (baseline + Relative) and absolute (baseline + Absolute) variations. The term “baseline + Delta features” signifies the inclusion of both baseline and Delta features in our analysis. In this study, we utilized ICARE and ML techniques such as KNN, Linear Discriminant Analysis (LDA), and Random Forest (RF) to predict relapse/progression. The performance of ICARE and ML techniques is compared for the progression prediction task. For the analysis TTP, we employed ICARE and CoxNet approaches to compare their performance. These prediction tasks are summarized in Table 2.

We also evaluated the possibility of predicting the recurrence volume on the EoT scans (n = 31) based on baseline PET-CT radiomics features (see Section 2.4.3).

#### 2.4.1. Progression Prediction

We used individual coefficient approximation for risk estimation (ICARE) classifier introduced by Rebaud et al. [47] for classification that employs a minimal learning strategy to mitigate the risk of overfitting when the dataset size is limited (that is the case in our current study). We also applied different machine learning techniques for progression prediction. Considering small datasets, utilizing a holdout or a very limited external dataset with similar characteristics is discouraged due to the significant uncertainty associated with a single small testing dataset [56]. As a preferable alternative, in this study we employed cross-validation (CV) after Z-score normalization to enhance reliability and robustness. We applied a nested CV with 10 outer folds and 5 repetitions, along with 5 inner folds and 20 repetitions on 85% of the dataset, and 15% of the dataset was used as unseen test data (after balancing the dataset). We evaluated the model with CV using accuracy and F1-score metrics and found that the scores were consistent across all the experiments. To balance the class labels in our feature set for both ICARE and ML approaches, we used the synthetic minority oversampling technique (SMOTE) algorithm to generate new data for the minority class. SMOTE is a technique used to generate additional samples from the minority class. Essentially, SMOTE creates a hypercube using instances from the feature space of malignant nodules and generates new samples by interpolating within that space, although there are some recent studies that showed SMOTE might not be very helpful [57], but it is still widely used in the machine learning pipelines.

For each ICARE model, a random selection of F features were made for the training process. These models were trained using their respective bootstrap samples from the train set, and then used to make predictions on the test set. Finally, the 1000 predictions from all the models were aggregated using the median. F is a hyperparameter of the ensemble model that should be selected to strike an appropriate balance between performance and efficiency. The ensemble model comprises three hyperparameters, namely F, $C_{min}$, and ρ. Specifically in this study, 1000 hyperparameter sets were randomly generated and assessed using Monte Carlo CV, and subsequently ranked based on their corresponding CV scores. During the testing phase, an ensemble model was trained using the selected B=5 hyperparameter sets, with each binary-weighted model randomly selecting a hyperparameter set from the chosen B. Finally, the B value was optimized using an additional CV step.

In our ML pipeline, after removing constant features and removing correlated (highly correlated with correlation threshold = 0.8) features, for feature selection, we used sequential feature selection (SFS), a wrapper method for feature selection, since in our preliminary studies it performed better compared to LASSO, RELIEF, linear/kernel-based PCA [58]. Random Forest (RF), KNN and Linear Discriminant Analysis (LDA) were used for progression prediction in this study.

#### 2.4.2. Time to Progression Survival Analysis

ICARE survival models (ICARESurvival and BaggedICARESurvival that uses the ensemble of predictions) predict a risk score, and the predictions are anti-concordant with the target [47]. We also used CoxNet Survival Analysis to predict TTP in this study. CoxNet, is a powerful statistical tool in survival analysis, building upon the foundation of Cox Regression. CoxNet incorporates LASSO (L1 regularization) to develop predictive models for survival outcomes, harnessing its ability to handle complex relationships within covariates while enhancing interpretability. By optimizing a carefully crafted objective function, CoxNet simultaneously minimizes the risk of overfitting while maintaining predictive accuracy.

The feature selection prior to applying survival analysis was done by SFS, Chi-Square, Mutual Information, Random Forest, and LASSO (filter based). The feature selection step is done using the binary outcome and the selected features were entered to the next step of CoxNet Survival Analysis. To perform hyperparameter tuning for CoxNet, the following steps are taken: After standardizing the input features, to fine-tune the model hyperparameters (*l1_ratio* (a regularization parameter), *alpha_min_ratio* (a minimum alpha ratio for regularization) and *max_iter* parameter), a 10-fold CV is set up with data shuffling and a random seed for reproducibility. A *GridSearchCV* was created to systematically search for the optimal alpha value that are used for regularization within the CoxNet Survival Analysis model.

For TTP analysis, we examined Baseline, EoT, Baseline + EoT, Relative Delta, Absolute Delta, Baseline + Relative Delta, and Baseline + Absolute Delta features. We also aimed to evaluate the predictive performance of EoT feature sets on a broader patient group, consisting of 50 cases for TTP assessment (see Table 2).

#### 2.4.3. Prediction of Recurrence (Subsequent) TMTV Values on EoT

We applied Gradient Boosting Regressor (GBR) to the PET-CT radiomics features of baseline scans (n = 31) to predict subsequent TMTV (recurrence volume) in the EoT scans of the corresponding patients. We first standardized the features and label (using *StandardScaler*). After removing highly correlated features, to perform hyperparameter tuning for GBR, a hyperparameter grid is defined, encompassing various combinations of hyperparameters, including the number of estimators, learning rate, maximum depth, minimum samples split, and minimum samples leaf. *GridSearchCV* was employed to perform a cross-validated grid search to find the optimal hyperparameters for the GBR. The best estimator (i.e., the model with the optimal hyperparameters) was selected from the results of the grid search. The performance of the best model was evaluated using several metrics, including R-squared (R^2^), Mean Absolute Error (MAE), and Mean Absolute Percentage Error (MAPE).

## 3. Results

In this section, we present the results of progression prediction (Section 3.1), TTP (Section 3.2), and recurrence volume prediction (Section 3.3), respectively.

### 3.1. Progression Prediction

First, we present the results of progression prediction using only EoT features for both PET and PET-CT, employing ICARE, KNN, LDA, and RF techniques on a larger group of cases (n = 50) (refer to Table 3). Then, we further analyze the progression prediction results using the ICARE technique with PET-only and PET-CT features on baseline, EoT, and delta radiomics, as well as the combination of baseline and delta radiomics (see Table 4 and Table 5). Table 6 demonstrates the comparison of machine learning techniques in assessing the predictive effectiveness of various feature combinations.

The results of the progression prediction using the radiomics features of EoT scans (n = 50) are shown in Table 3. LDA and RF performed better on PET and PET-CT features, respectively. The selected features using SFS performed better compared to LASSO, RELIEF and PCA (Supplemental Table S1). Other metrics are presented in Supplemental Table S2 for comparison. The hierarchical clustering of EoT PET-CT features and the corresponding Kaplan–Meier (KM) plots using the phenotype suggested by unsupervised clustering are shown in Figure S2. This analysis demonstrated the discriminative power of our supervised radiomics signature.

Table 4 and Table 5 provide a concise overview of the CV results of progression prediction with Bagged ICARE based on baseline, EoT, baseline + EoT, Delta (relative), Delta (absolute), baseline + Delta (relative), baseline + Delta (absolute) of PET features and PET-CT features. The overall performance using PET-CT features is higher compared to PET only features for relapse/progression prediction. Based on Table 5, EoT features seems to be a feature set that performed relatively well in terms of accuracy, as it has a higher mean accuracy compared to other single time feature sets (baseline). Absolute and baseline + Relative performed better in terms of accuracy.

Table 6 illustrates the comparison of ML techniques in evaluating the predictive efficacy of feature combinations that demonstrated strong performance compared to ICARE (Table 5). In the EoT feature set, RF achieves the highest accuracy and F1 score among the three algorithms. It also has relatively low standard deviations, suggesting stable performance. The baseline + Relative feature set shows high accuracy and F1 Score for KNN, Absolute Delta for LDA, and Relative Delta for RF. Other metrics are presented in Supplemental Table S3 for comparison.

### 3.2. TTP Analysis

For TTP analysis, we used ICARE and CoxNet. We applied different feature selection methods for CoxNet. The best results of TTP analysis are shown in Table 7. Among all the feature sets and their combinations, the prediction model based on baseline + Delta features and the EoT PET-CT features performed better in terms of concordance index (Harrell’s c-index) using CoxNet. ICARE showed limited efficacy in improving task TTP based solely on baseline features (with a c-index < 0.4). However, when incorporating both baseline + Delta and EoT features, its performance notably improved. In both categories of features, the c-indices of CoxNet technique applied to the selected feature by sequential feature selection and Chi-Square, Mutual Information, Random Forest, and LASSO.

The predictive performance of the DS for relapse/progression was assessed, yielding a c-index of 0.66 ± 0.09 for a sample size of n = 50 and 0.67 ± 0.03 for n = 31 cases. Notably, no significant differences were observed when compared to the best radiomics signature of the EoT scans (n = 31) and baseline combined with Delta features (*p*-value > 0.05). The current results do not allow us to draw a conclusion regarding the statistical significance of the superior predictive performance of EoT features on a larger sample size (n = 50) compared to a subset (n = 31) when assessing TTP. In Table 7, the bold values highlight the technique that demonstrated superior performance within each feature set (row).

### 3.3. Prediction of Recurrence Volume on EoT Scans

Table 8 represents the results of recurrence volume prediction using GBR. An R2 score of 0.86 indicates that the GBR model explains 86% of the variance in the target variable. An MAE of 0.29 suggests relatively good accuracy in GBR predictions. A MAPE of 0.39 suggests that, on average, GBR predictions are off by about 39% from the actual values.

## 4. Discussion

A significant challenge in the clinical management of PMBCL is recognizing patients who are unlikely to respond well to the initial treatment (chemotherapy) [22]. The identification of patients at risk of progression or relapse in lymphoma is currently limited by single time-point assessments, such as the International Prognostic Index (IPI) based on baseline and DS based on EoT scans. For EoT evaluation, DS serves as a key criterion, but its simplicity and ambiguity in defining DS = 4 and DS = 5 necessitate more precise prognostic markers. Therefore, precisely identifying predictive biomarkers is crucial for distinguishing patients who stand to gain from an initially intensive treatment approach while also avoiding unnecessary interventions for those with a high probability of favorable outcomes. Research has suggested that high heterogeneity in the distribution of [^18^F]FDG uptake within a tumor on PET/CT scans could be a potential marker of chemoresistance in solid tumors [10]. While some studies have investigated Delta SUVmax in DLBCL [59,60], to the best of our knowledge, no published studies have assessed changes overtime in radiomic features for PMBCL relapse/progression prediction.

Determining the interpretability of radiomics features is somewhat subjective; for instance, features linked to tumor shape can be easily comprehended by non-experts, while those rooted in voxel grey-level distributions necessitate specialist knowledge, and texture-related features are challenging to understand, even for image processing experts [61]. Based on our findings, (Table S1), the most predictive Delta radiomics features in our study are: PET features including GLSZM (Normalized grey level non-uniformity), GLCM (difference average, correlation, normalized inverse difference moment), GLRLM (grey level non-uniformity), Intensity histogram (root mean square and uniformity), local intensity based (coefficient of variation, intensity peak discretized volume sought), CT features including GLSZM (zone size entropy), GLCM (Sum Entropy, Zone size entropy), local intensity based (intensity peak discretized volume sought), and the innermost RIM features in PET image. The analysis revealed that specific sub-regions closer to the center of the tumor were most predictive in PET images, and helped the radiomics signature to improve the prediction performance. While a combination of sub-regions near the core and boundaries were significant in CT images, although not as predictive as other Delta radiomics features when we considered them together. We also showed that the hierarchical clustering of EoT PET-CT features, using the phenotype suggested by unsupervised clustering did not perform well (Figure S1).

Given changes in scanner types across different time points in some cases, we meticulously evaluated the additive and multiplicative impact of these variations on radiomics features. The analysis revealed no discernible effect, indicating that harmonization was unnecessary. However, we implemented longitudinal harmonization (Long ComBat) that is designed for feature harmonization in longitudinal studies [55]. To assess the statistical difference between two sets of feature values after harmonization the Kolmogorov–Smirnov test was applied. The results, presented in the Supplementary Material (Figure S3), confirm that harmonized features showed no significant differences when compared to non-harmonized features. Consequently, the features that we used in this study are not harmonized.

Progression/relapse prediction using ICARE and ML on baseline, EoT, Baseline + EoT, Delta (relative), Delta (absolute), baseline and Delta (relative) and baseline + Delta (Absolute) showed that Delta PET-CT features outperformed both baseline features and the use of PET features alone in predicting progression showed that CT features improve the performance of the prediction tasks. ML techniques, especially RF and LDA (for absolute and relative Delta) showed superior performance compared to ICARE.

To evaluate the effect of bias due to the limited dataset, we started our analysis from the EoT scans that showed comparable performance for progression prediction on the data with the same size (n = 31) and better performance on a bigger dataset (n = 50) (Table 3). Our analysis of progression/relapse prediction using EoT features of 50 cases, including PET and PET-CT, revealed that the predictive capability of PET-CT features significantly outperformed PET-only features in a larger dataset too when employing ICARE and RF for prediction, as indicated by higher F1 scores and accuracy rates (Table 3). Furthermore, when applied to the larger group comprising 50 cases with EoT PET-CT features, the performance of relapse/progression prediction using the RF technique exceeded that observed in the smaller subset with 31 EoT scans, with an accuracy of 0.92 ± 0.02 (Table 3) compared to 0.83 ± 0.05 (Table 6), as well as the ICARE technique with accuracies of 0.79 ± 0.09 (Table 3) and 0.78 ± 0.14 (Table 5), respectively. However, these results did not exhibit significant statistical differences.

For TTP prediction based on baseline + absolute Delta radiomics on 31 cases, CoxNet with LASSO and SFS, with respective c-indices of 0.67 ± 0.06 and 0.65 ± 0.17 performed better compared to ICARE (c-index = 0.61 ± 0.11). The TTP analysis conducted on the 50 cases also demonstrated the superior performance of CoxNet (LASSO) methodology (Table 7). TTP prediction on 50 cases based on EoT PET-CT radiomics, as it is shown in Table 7, CoxNet with SFS and LASSO, with respective c-indices of 0.68 ± 0.09 and 0.67 ± 0.09 performed better compared to ICARE (c-index = 0.65 ± 0.23) (*p*-value < 0.05). However, the current findings do not permit a conclusive determination regarding the statistical significance of the enhanced predictive capability of EoT features on a larger sample size (n = 50) versus a subset (n = 31) in TTP assessment.

In this study, in all the prediction analyses, we considered various segmentation approaches, including 41% SUVmax, 25% SUVmax, manual contouring, and PET-Edge by the MIM software. However, no significant differences were observed in terms of performance metrics across any of the tasks using ICARE and ML approaches. In addition, our results for recurrence volume (TMTV) prediction showed that GBR is able to predict the recurrence volume in EoT scans based on the radiomics features of the baseline scans with MAE error of 0.29 ± 0.12. It is important to note that the objective of this experiment is to predict recurrence volume on future scan (EoT) based on baseline radiomics features. The effectiveness of this approach requires further investigation with an extensive set of training data.

Our study bears several limitations. Firstly, we conducted a retrospective analysis on patients with both baseline and EoT scans, along with corresponding expert manual segmentation for Delta radiomics analysis. This process adhered to specific inclusion criteria, potentially introducing selection bias. Secondly, the sample size of the patient group was relatively small and monocentric (a single institution) but with two scanners. To prevent misinterpretation due to overfitting, it is vital to assess model performance on data distinct from the training set [62]. This dataset is termed a holdout test set or external validation. While a substantial external holdout test set is ideal for generalization, it is not always available. In such cases, (nested) CV is frequently employed to estimate generalization performance, but to enhance the generalizability of our suggested radiomics model(s), future validation on larger patient groups of PMBCL cases within multi-centric settings, facilitating improved reliability and reproducibility. It should be noted that early FDG PET-CT during treatment was not evaluated in this study, which is another limitation to address in future studies.

We also considered the generalizability of our trained ICARE and ML models on progression prediction of DLBCL cases (n = 50) based on their EoT scans, but the results showed these models trained on our PMBCL dataset are not generalized well on DLBCL cases (Accuracy = 0.34, F1-score = 0.38). Extended evaluation on DLBCL cases is beyond the scope of this paper and will be presented in another study. In our prediction approaches, we aggregated all the lesions, aiming to calculate a general tumor spatial characteristic for each patient. Our PMBCL cases mostly have a single lesion but for DLBCL cases with multiple lesions we should consider the weighted approach or the largest or hottest lesion. This assumption is based on empirical observations which suggest that the largest lesion commonly exhibits the most rapid growth and predominantly influences overall survival (OS) outcomes. It might be one of the considerations that should be taken into account for generalizability evaluations. Moreover, investigating the usefulness of incorporating clinical factors such as blood test parameters could be beneficial (i.e., ctDNA [63]).

Based on the comprehensive analysis conducted in this study, we conclude that ML approaches outperformed ICARE in both the tasks of predicting progression and TTP. In addition, our investigation into the predictive efficacy of Delta radiomics unveils results that align closely with the predictive capabilities of EoT radiomics. When applied to a larger group of 50 cases with EoT PET-CT features, the RF and ICARE techniques demonstrated improved performance in relapse/progression prediction compared to the smaller subset of 31 EoT scans (*p*-value < 0.05). TTP prediction is also enhanced when utilizing the EoT PET-CT features in the larger dataset (n = 50). However, these differences for TTP on the datasets with the size of 31 and 50 did not reach statistical significance. This suggests that, within the context of our specific dataset, Delta radiomics holds comparable predictive significance to EoT radiomics (Table 6).

## 5. Conclusions

In this feasibility study, we explored the use of Delta radiomics to evaluate changes after treatment which could predict progression/relapse and TTP in a group of PMBCL patients using ICARE and ML techniques on the baseline, EoT, and Delta (relative and absolute) feature combinations. We found that Delta PET-CT features and its combination with baseline features outperformed baseline and EoT features in predicting relapse/progression, with ML techniques, particularly RF and LDA, showing superior performance compared to ICARE. Specifically, our study demonstrated that for predicting relapse/progression using Delta radiomics between baseline and EoT PET-CT scans, ICARE, RF, and LDA achieved the highest performances in terms of accuracy and F1 score. Specifically, ICARE achieved an accuracy of 0.81 ± 0.15 and an F1 score of 0.77 ± 0.18, while RF and LDA achieved accuracies of 0.89 ± 0.04 and 0.89 ± 0.03, respectively, with corresponding F1 scores of 0.87 ± 0.04 and 0.89 ± 0.03. These results indicate superior predictive power of Delta radiomics compared to using only EoT radiomics features. Moreover, in terms of TTP prediction, the combination of baseline and Delta (absolute) radiomics outperformed both baseline and EoT PET-CT features when utilizing CoxNet with LASSO feature selection. Such promising findings may enhance clinical decision-making at an earlier time-point to enhance patient outcomes and limit potential toxicity. Future research using larger groups of patients with other lymphoma sub-types should provide external validation regarding the utility of Delta radiomic feature analysis in clinical practice as a key component of lymphoma management. The study underscores the importance of EoT scans and the potential of Delta radiomics in predicting disease progression/relapse in PMBCL patients based on [^18^F]FDG PET-CT scans.

## Figures and Tables

**Table 1:** Summary of the data in this study (EoT: End of treatment, std: standard deviation).

Scan Time-Point	# of Cases	Progression (Percentage)	Average Follow-Up (y) (±std)
EoT	50	24.0%	5.56 ± 3.74
Baseline and EoT	31	15.6%	3.73 ± 2.17

**Table 2:** Our analysis on different features in this study. EoT: End of Treatment, RF: Random Forest, KNN: K-Nearest Neighbors, LDA: Linear Discriminant Analysis. The usage of ‘+’ in “baseline + Delta features” refers to the inclusion of both baseline and Delta features in our analysis.

Number of Cases	Scan Time Points	Features Set (PET and CT)	Task		Prediction Approach	
31	Single time point scan	baseline	Progression prediction (Section 2.4.1)	Time to Progression (Section 2.4.2)	ICARE (Individual Coefficient Approximation for Risk Estimation) [47]	Machine Learning (RF, KNN, LDA)
		EoT				
31	Two time points scans	baseline + EoT				
31	Delta	Relative				
		Absolute				
31	Baseline + Delta	baseline + Relative				
		baseline + Absolute				
50	Single time point scan	EoT				

**Table 3:** The performance of relapse/progression prediction, achieved through the application of ICARE and machine learning approaches on radiomics features extracted from End-of-Treatment (EoT) scans (n = 50). (ICARE: Individual coefficient approximation for risk estimation, KNN: K-Nearest Neighbor, LDA: Linear Discriminant Analysis, RF: Random Forest). Bold stands for statistically significant differences between PET EoT and PET-CT EoT features (comparing rows in each column) (*p*-value < 0.05).

Features	ICARE		KNN		LDA		Random Forest	
	Accuracy	F1 Score	Accuracy	F1 Score	Accuracy	F1 Score	Accuracy	F1 Score
PET EoT	0.61 ± 0.07	0.56 ± 0.12	0.80 ± 0.03	0.83 ± 0.02	0.85 ± 0.04	0.85 ± 0.04	0.87 ± 0.03	0.87 ± 0.03
PET-CT EoT	**0.79 ± 0.09**	**0.81 ± 0.08**	0.81 ± 0.02	0.84 ± 0.02	0.85 ± 0.02	0.86 ± 0.02	**0.92 ± 0.02**	**0.91 ± 0.02**

**Table 4:** The results of relapse/progression prediction using ICARE based on baseline, End of treatment (EoT), baseline + EoT, Delta (relative), Delta (absolute), baseline + Delta (relative) and baseline + Delta (absolute) from PET images, the results of cross-validation. (Bold stands for statistically significant differences between feature sets (in each columns the rows were compared) (*p*-value < 0.05)). The usage of ‘+’ refers to the inclusion of both baseline and Delta features in our analysis.

Features Set (PET)	Accuracy	F1 Score	Recall	Precision	ROC AUC
Baseline	0.56 ± 0.16	0.57 ± 0.19	0.58 ± 0.12	0.65 ± 0.15	0.77 ± 0.14
EoT	0.56 ± 0.18	0.56 ± 0.18	0.47 ± 0.14	0.62 ± 0.12	0.63 ± 0.10
Baseline + EoT	0.64 ± 0.12	0.67 ± 0.14	0.59 ± 0.13	0.63 ± 0.13	0.77 ± 0.13
Relative Delta	0.65 ± 0.18	0.67 ± 0.19	0.53 ± 0.11	0.61 ± 0.11	0.67 ± 0.15
Absolute Delta	**0.69 ± 0.25**	0.66 ± 0.24	0.55 ± 0.14	0.56 ± 0.06	0.63 ± 0.19
Baseline + Relative Delta	0.65 ± 0.16	0.67 ± 0.14	**0.63 ± 0.15**	0.63 ± 0.13	**0.81 ± 0.08**
Baseline + Absolute Delta	0.64 ± 0.19	0.63 ± 0.21	0.58 ± 0.12	0.59 ± 0.09	0.69 ± 0.14

**Table 5:** The results of relapse/progression prediction using ICARE based on baseline, End of treatment (EoT), Baseline + EoT, Delta (relative), Delta (Absolute), baseline and Delta (relative) and baseline + Delta (Absolute) from PET-CT images, the results of cross-validation. ((Bold stands for statistically significant differences between feature sets (in each columns the rows were compared) (*p*-value < 0.05)). The usage of ‘+’ refers to the inclusion of both baseline and Delta features in our analysis.

Features Set (PET-CT)	Accuracy	F1 Score	Recall	Precision	ROC AUC
Baseline	0.60 ± 0.16	0.65 ± 0.15	0.58 ± 0.24	0.66 ± 0.16	0.79 ± 0.09
EoT	0.78 ± 0.14	0.76 ± 0.19	0.50 ± 0.14	0.75 ± 0.25	0.75 ± 0.12
Baseline + EoT	0.69 ± 0.17	0.65 ± 0.31	**0.68 ± 0.24**	0.75 ± 0.25	**0.88 ± 0.13**
Relative Delta	0.66 ± 0.16	0.60 ± 0.19	0.62 ± 0.14	0.71 ± 0.21	0.88 ± 0.14
Absolute Delta	0.81 ± 0.15	0.77 ± 0.18	0.63 ± 0.15	0.68 ± 0.18	0.87 ± 0.17
Baseline + Relative Delta	**0.84 ± 0.11**	**0.82 ± 0.13**	0.50 ± 0.14	0.75 ± 0.25	0.75 ± 0.15
Baseline + Absolute Delta	0.70 ± 0.13	0.60 ± 0.24	0.57 ± 0.13	0.75 ± 0.25	0.74 ± 0.09

**Table 6:** The results of relapse/progression prediction based on End of treatment (EoT), Delta (relative), Delta (absolute), and baseline + Delta (relative) for PET-CT scans, the results of CV with machine learning (ML) pipeline. KNN: K-Nearest Neighbor, LDA: Linear Discriminant Analysis, RF: Random Forest. Bold stands for statistically significant differences between ML techniques (in each column, the rows were compared) (*p*-value < 0.05). The usage of ‘+’ refers to the inclusion of both baseline and Delta features in our analysis.

Features Set (PET-CT)	KNN		LDA		Random Forest	
	Accuracy	F1 Score	Accuracy	F1 Score	Accuracy	F1 Score
Baseline	0.67 ± 0.14	0.65 ± 0.13	0.68 ± 0.10	0.69 ± 0.09	0.69 ± 0.18	0.69 ± 0.04
EoT	0.78 ± 0.04	0.77 ± 0.04	0.77 ± 0.02	0.74 ± 0.03	0.83 ± 0.05	0.84 ± 0.05
Relative Delta	0.77 ± 0.05	0.82 ± 0.03	0.82 ± 0.03	0.80 ± 0.3	0.89 ± 0.04	0.87 ± 0.05
Absolute Delta	0.73 ± 0.02	0.77 ± 0.02	**0.89 ± 0.03**	**0.89 ± 0.03**	0.87 ± 0.04	0.86 ± 0.05
Baseline + Relative Delta	**0.86 ± 0.03**	**0.87 ± 0.03**	0.75 ± 0.04	0.75 ± 0.04	0.88 ± 0.03	0.87 ± 0.03

**Table 7:** Concordance Index (c-index) for Time-to-progression analysis (EoT: End of Treatment, Bold stands for statistically significant differences between feature sets (in each row comparing different columns) (*p*-value < 0.05)). The usage of ‘+’ refers to the inclusion of both baseline and Delta features in our analysis. MI: Mutual Information, RF: Random Forest.

Features Set (PET-CT)	#Cases	ICARE	CoxNet (SFS)	CoxNet (Chi-Square)	CoxNet (MI)	CoxNet (RF)	CoxNet (LASSO)
Baseline + Delta (absolute)	31	0.61 ± 0.11	0.65 ± 0.17	0.60 ± 0.08	0.60 ± 0.04	0.57 ± 0.10	**0.67 ± 0.06**
EoT	31	0.60 ± 0.24	0.54 ± 0.04	0.45 ± 0.10	0.37 ± 0.10	0.58 ± 0.09	**0.65 ± 0.07**
EoT	50	0.65 ± 0.23	**0.68 ± 0.09**	0.56 ± 0.03	0.64 ± 0.05	0.58 ± 0.04	0.67 ± 0.09

**Table 8:** Gradient Boosting Regressor performance for recurrence volume prediction.

PET-CT Features Set (n = 31 Cases)	Gradient Boosting Regressor	R-squared (R^2^)	Mean Absolute Error	Mean Absolute Percentage Error
Baseline	Tuned with Grid Search	0.86 ± 0.09	0.29 ± 0.12	0.39 ± 0.15

## Data Availability

To safeguard our patients’ information, we are unable to disclose the data. However, for inquiries regarding the radiomics features, kindly reach out to the corresponding author.

## Supplementary Materials

The following supporting information can be downloaded at: https://www.mdpi.com/article/10.3390/cancers16061090/s1, Table S1: Selected features of EoT and Delta radiomics using ICARE and different feature selection approaches; Table S2: The results of progression prediction based on cases that only have EoT scans (n = 50), (* *p*-value < 0.05); Table S3: The results of progression prediction based on End of treatment (EoT), Delta (relative), Delta (absolute), and baseline + Delta (relative) from PET-CT images; Figure S1: Hierarchical clustering of EoT PET-CT features; Figure S2: Kaplan–Meier plots for phenotype features that were clustered by an unsupervised approach (Figure S2) and for our suggested radiomics signature (predicted by Random Forest technique). Log-rank test *p*-value < 0.05; Figure S3: The longitudinal harmonization effect on some of the predictive features.
