# Supplementary material for: Evaluating Outcome Prediction via Baseline, End-of-Treatment, and Delta Radiomics on PET-CT Images of Primary Mediastinal Large B-Cell Lymphoma
Source: Cancers (Basel). 2024 Mar 8;16(6):1090. doi: 10.3390/cancers16061090 (PMC10968861; doi:10.3390/cancers16061090)
Supplement: Supplementary file 1 [file cancers-16-01090-s001.zip › cancers-2895775-supplementary.pdf]

Supplemental Material

Complementary analysis and plots

The predicative features by ICARE and the selected features by SFS for ML approaches are shown in Table S1.

Table S1. Selected features of EoT and Delta radiomics using ICARE and different feature selection approaches

Techniques	ICARE	SFS	LASSO	Random Forest
Selected features (EoT)	MTV<br>NGTDM<br>GLRLM<br>GLSZM<br>INTENSITY<br>GLRLM<br>GLCM<br>SUV<br>RIM	MTV<br>NGTDM<br>GLRLM<br>GLSZM<br>GLCM<br>RIM<br>SUV	MTV<br>NGTDM<br>GLRLM<br>GLSZM<br>LOCAL INTENSITY<br>RIM	MTV<br>INTENSITY<br>Maximum Histogram Gradient Grey Level(HU)<br>SUV<br>RIM
Selected features (Delta)	GLSZM<br>GLCM<br>GLRLM<br>Intensity (coefficient of variation, intensity peak discretized volume sought)<br>RIM	GLCM<br>GLSZM<br>NGTDM<br>GLRLM<br>RIM<br>AUC CSH<br>INTENSITY(Histogram Kurtosis)	GLCM<br>GLSZM<br>NGTDM<br>GLRLM<br>INTENSITY (coefficient of variation, coefficient of dispersion)<br>Skewness	GLCM<br>GLSZM<br>RIM<br>AUC CSH

Table S2. The results of progression prediction based on cases that only have EoT scans (n=50), (*p-value<0.05)

Features	ICARE			KNN			LDA			Random Forest		
	Recall	Precision	ROC AUC	Recall	Precision	ROC AUC	Recall	Precision	ROC AUC	Recall	Precision	ROC AUC
PET EoT	0.52±0.11	**0.65±0.15**	0.70±0.09	0.47±0.13	0.68±0.16	0.70±0.19	**0.62±0.15**	0.60±0.26	0.74±0.05	0.57±0.14	0.72±0.19	0.88±0.09
PETCT EoT	**0.61±0.14**	0.64±0.14	**0.80±0.14***	**0.62±0.16***	**0.76±0.17***	**0.86±0.09***	0.55±0.16	**0.83±0.24***	**0.99±0.01***	**0.60±0.15***	**0.77±0.18***	**0.90±0.11***

Table S3. The results of progression prediction based on End of treatment (EoT), Delta (relative), Delta (absolute), and baseline + Delta (relative) from PET-CT images.

Features set (PETCT)	KNN			LDA			Random Forest		
	Recall (sensitivity)	Precision	ROC AUC	Recall	Precision	ROC AUC	Recall	Precision	ROC AUC
EoT	0.56±0.15	0.60±0.14	0.78±0.12	0.56±0.15	0.63±0.24	0.99±0.14	0.55±0.10	0.68±0.22	**0.99±0.08**

Relative Delta	0.62±0.18	**0.75±0.21**	**0.88±0.08**	0.50±0.10	0.43±0.13	0.75±0.08	**0.62±0.18**	**0.75±0.21**	0.87±0.11
Absolute Delta	**0.67±0.17**	0.65±0.15	0.88±0.11	**0.67±0.17**	0.65±0.15	0.88±0.13	0.61±0.14	0.75±0.25	0.92±0.13
Baseline + Relative	**0.67±0.17**	**0.75±0.25**	0.77±0.09	**0.67±0.17**	**0.75±0.25**	**1.00±0.09**	0.50±0.12	0.70±0.21	0.81±0.14

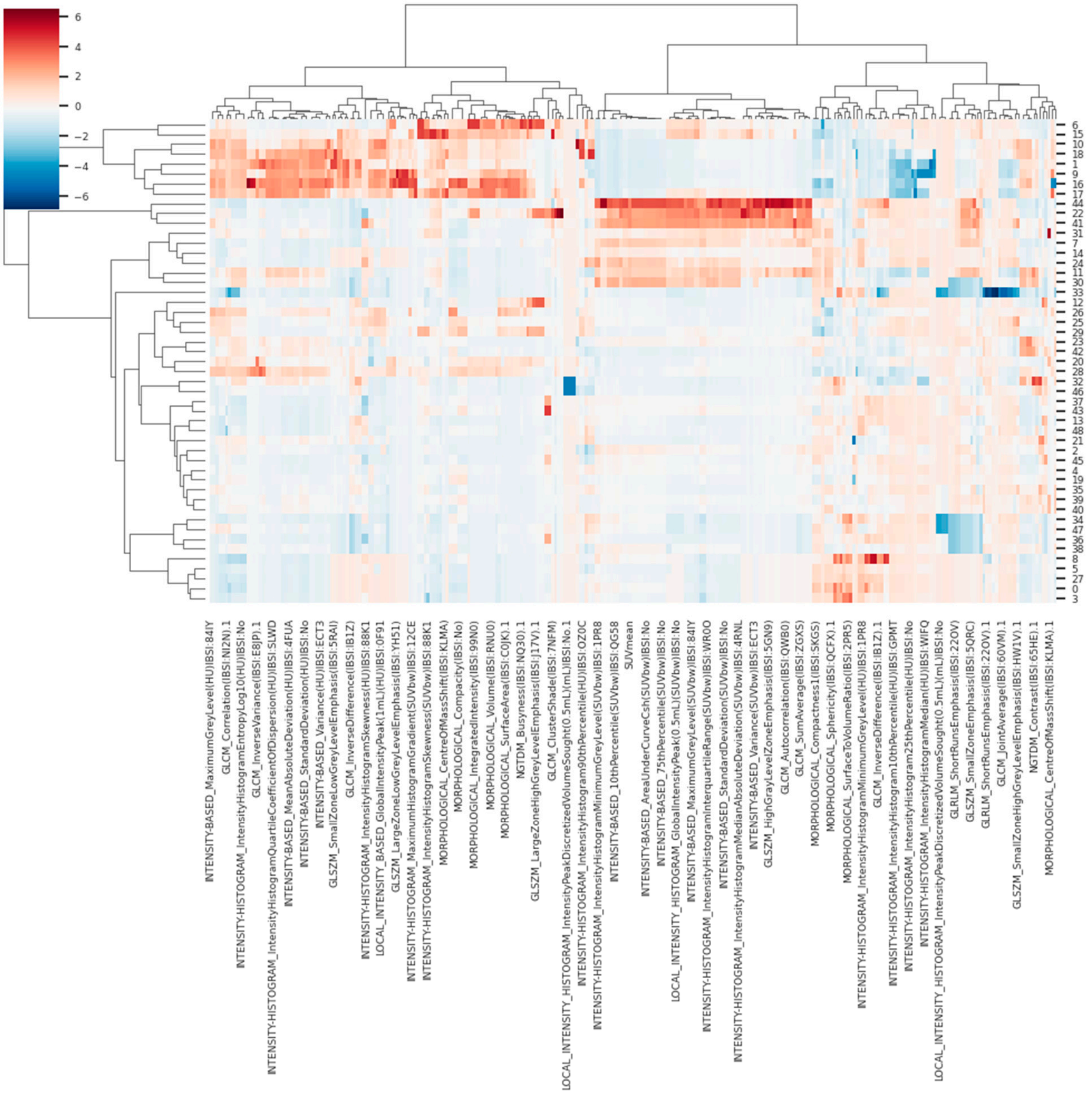

Figure S1. Hierarchical clustering of EoT PET-CT features

Hierarchical clustering of EoT PET-CT features, using the phenotype suggested by unsupervised clustering did not perform well. The following KM plots showed the discriminative power of our suggested radiomics signature.

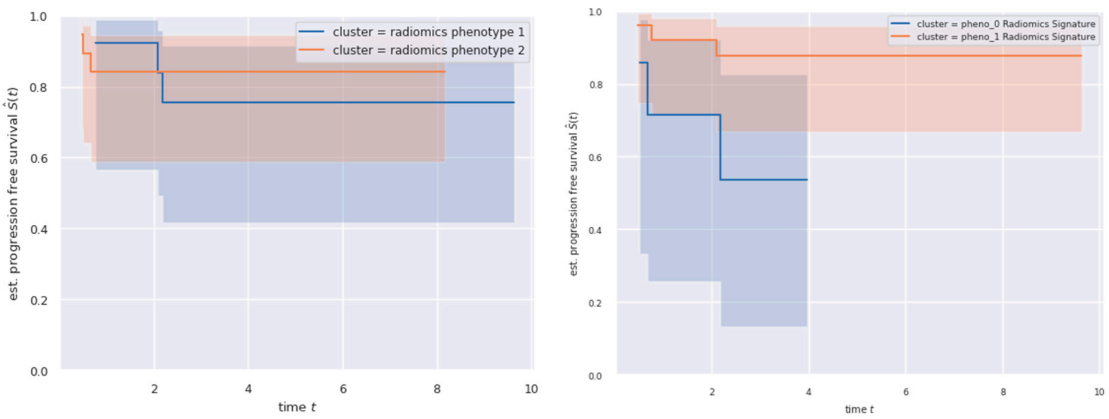

Figure S2. Kaplan-Meier plots for phenotype features that were clustered by an unsupervised approach (Figure S2) and for our suggested radiomics signature (predicted by Random Forest technique). Log-rank test p-value <0.05.

Harmonization effect

We first applied the two-sample t-test and Bartlett's test respectively for mean shift (additive effect) and heteroscedasticity (multiplicative effect) assessment and the results of both test were not significant. However, we applied longitudinal harmonization (Long ComBat) to see if the features would change significantly. The null hypothesis is not rejected meaning that the two sets of feature values before and after harmonization are not significantly different.

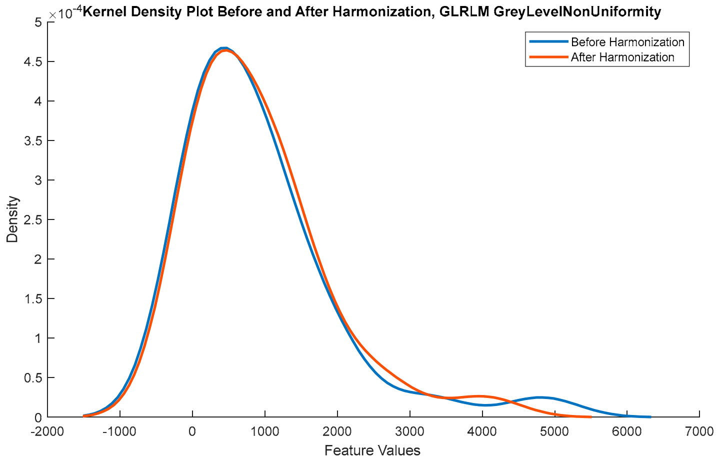

(a) Kolmogorov-Smirnov test statistic: 0.1875, p-value: 0.58

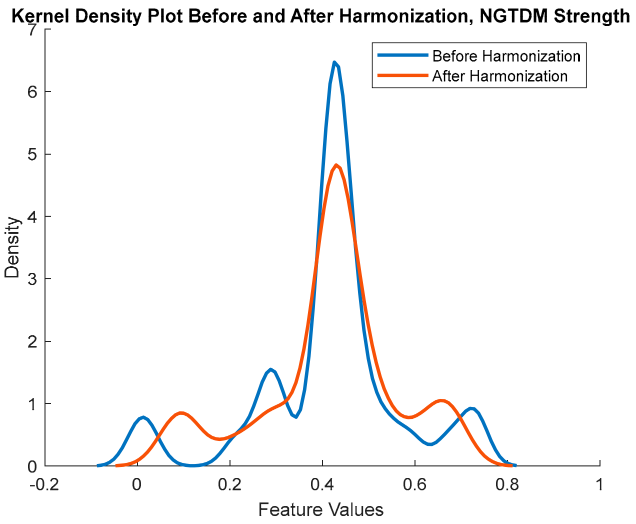

(b) Kolmogorov-Smirnov test statistic: 0.16, p-value: 0.80

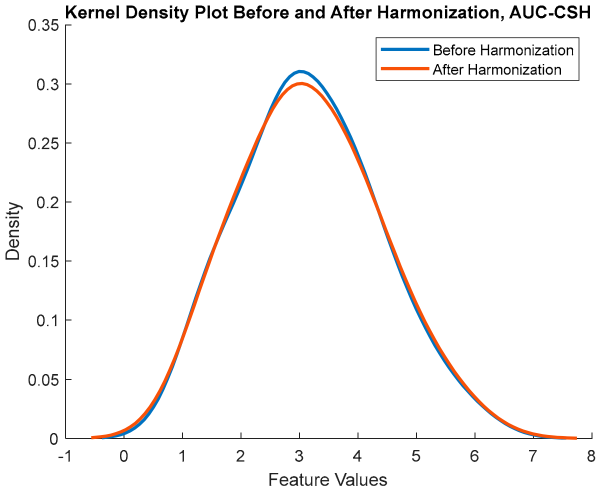

(c) Kolmogorov-Smirnov test statistic: 0.25, p-value: 0.23

Figure S3. The longitudinal harmonization effect on some of the predictive features
